# Prognostic significance of combined α-fetoprotein and CA19-9 for hepatocellular carcinoma after hepatectomy

**DOI:** 10.1186/s12957-022-02806-9

**Published:** 2022-10-19

**Authors:** Jie Zhang, Shang Dong Qin, Yan Li, Fei Lu, Wen Feng Gong, Jian Hong Zhong, Liang Ma, Jing Fei Zhao, Guo Hua Zhan, Peng Zhan Li, Bin Song, Bang De Xiang

**Affiliations:** 1 Department of Hepatobiliary Surgery, Guangxi Medical University Cancer Hospital, Guangxi, China; 2 Guangxi Medical Information Institute, Guangxi, China; 3 Guangxi Medical University, Guangxi, China

**Keywords:** Hepatocellular carcinoma, Hepatectomy, α-fetoprotein, CA19-9, Prognosis

## Abstract

**Background:**

The prognosis of hepatocellular carcinoma (HCC) varies considerably among patients with the same disease stage and characteristics, and only about two thirds show high levels of α-fetoprotein (AFP), a common prognostic indicator for HCC. Here, we assessed whether the combination of presurgical serum levels of AFP and carbohydrate antigen 19-9 (CA19-9) can predict the prognosis of HCC patients after hepatectomy.

**Methods:**

The clinicopathological characteristics and post-hepatectomy outcomes of 711 HCC patients were retrospectively reviewed. The patients were classified into three groups based on whether their preoperative serum levels of both AFP and CA19-9 were higher than the respective cut-offs of 400 ng/ml and 37 U/ml [double positive (DP)], the level of only one marker was higher than the cut-off [single positive (SP)], or neither level was higher than the cut-off [negative (N)]. The overall survival (OS) and recurrence-free survival (RFS) rates were estimated using Kaplan–Meier curves. Univariate and multivariate survival analyses were performed to identify the clinicopathological factors significantly associated with HCC prognosis.

**Results:**

The 1-year, 3-year, and 5-year RFS and OS rates in the N group were significantly higher than those in the SP group, while the DP group showed the lowest rates. Multivariate Cox regression analysis showed that large tumor size (> 5 cm), multiple tumors (≥ 2), incomplete tumor capsule, positive microvascular invasion, Barcelona Clinic Liver Cancer C stage, and CA19-9 level > 37 U/mL were independent risk factors for RFS and OS in HCC patients. Moreover, aspartate aminotransferase levels > 40 U/L proved to be an independent prognostic factor for OS.

**Conclusion:**

The combination of serum AFP and CA19-9 levels may be a useful prognostic marker for HCC patients after hepatectomy.

## Introduction

Hepatocellular carcinoma (HCC) is one of the most common malignancies in the world and is the third most common cause of cancer mortality [1]. Surgical resection is the most effective way to treat HCC [2]. However, the 5-year recurrence rate after hepatectomy is up to 40–70% [3], indicating the need for further investigation of the prognostic characteristics of HCC.

α-fetoprotein (AFP) is a widely known prognostic marker of HCC used to identify high-risk populations [4]. Our previous long-term follow-up study of 396 HCC patients confirmed that high preoperative levels of AFP lead to poor prognosis of HCC after hepatectomy [5]. However, AFP levels are elevated in only about two-thirds of patients diagnosed with HCC [4], implying that the AFP cut-off value of >400 ng/mL in European guidelines [6] cannot reliably predict HCC prognosis on its own.

Carbohydrate antigen 19-9 (CA19-9), also known as sialyl-Lewis A antigen, is a tumor marker commonly used to screen different human cancers in the digestive system [7]. High serum CA19-9 levels in pancreatic, colorectal, and gastric tumors are associated with poor prognosis [8–10]. We recently found that CA19-9 levels >37 ng/mL correlate with poor prognosis of HCC [11].

Although CA19-9 and AFP are used in clinical diagnosis as single markers and their prognostic value in HCC has been demonstrated. A cohort study of 304 HCC patients obtained a significant cut-off value of CA19-9 of 27U/mL according to the AUC curve, and its multivariate analysis showed that a preoperative CA19-9 value >27 U/mL was independent prognostic factors for long-term survival and associated with poor prognosis after resection for HCC [12]. In another study on the relationship between preoperative serum AFP level and prognosis of HCC patients, 20 ng/mL and 400 ng/mL were selected as cut-off values, and the results showed that preoperative serum AFP level has considerable predictive value for the malignant feature and a preoperative AFP value >400 ng/mL was independent prognostic factors for long-term survival and associated with poor prognosis after resection for HCC [13]. The efficiency of their combination as a prognostic factor for HCC patients after hepatectomy has not yet been reported, except for a retrospective study of only 67 patients [14]. Therefore, here, we aimed to retrospectively compare the prognostic value of CA19-9 and AFP levels, individually and together, in HCC patients after hepatectomy.

## Methods

### Study population and data collection

A total of 921 consecutive patients with histologically proven HCC who underwent hepatectomy in the Department of Liver Surgery at Guangxi Medical University Cancer Hospital (Nanning, China) from January 2014 to December 2016 were considered for this study.

Clinicopathological data of the enrolled patients had been entered prospectively into the hepatectomy database of the Department of Liver Surgery at Guangxi Medical University Cancer Hospital, and they were retrospectively reviewed in the present study. The tumor size was considered as the maximum diameter of the largest tumor in the resected specimens. The collected tumors were histopathologically differentiated as grade I (well differentiated), grade II (moderately differentiated), or grade III–IV (poorly differentiated) according to the Edmondson–Steiner criteria. Preoperative AFP and CA19-9 levels were extracted from the hospital database. For most patients, both values were obtained within seven days prior to surgery.

### Hepatectomy

All surgical procedures were performed by experienced surgeons using standard techniques in the Department of Liver Surgery. All patients underwent curative resection, defined as the removal of all tumor lesions based on macroscopic inspection and a negative histology resection margin, as well as the absence of residual tumor or portal tumor thromboses during postoperative imaging. At this stage, patients were excluded if they were found to have other malignancies, distant metastasis, or lymph node involvement. The study was approved by the Ethics Committee of the Guangxi Medical University Cancer Hospital (LW2021056).

### Postoperative treatment

HCC patients received further treatment after hepatectomy to reduce recurrence risk. In particular, postoperative prophylactic transcatheter arterial chemoembolization was performed in patients with portal vein tumor thrombus or high-risk macrovascular invasion. Patients chronically infected with hepatitis B virus received long-term antiviral therapy with nucleoside analogs (entecavir or tenofovir). In the event of recurrence, repeat resection, radiofrequency ablation, transcatheter arterial chemoembolization, radiotherapy, or systemic treatment were applied depending on the patient’s condition and characteristics of the recurrence.

### Follow-up

All patients were followed up at our outpatient clinic or by telephone interview one month after surgery, then every three months for the rest of the first year, and every six months thereafter. The follow-up period ranged from 1 to 63 months with an average of 33 months, and it lasted until April 2019. The follow-up visits included a physical examination, liver function tests, measurement of serum AFP and CA19-9 levels, abdominal ultrasonography, and computed tomography or magnetic resonance imaging.

Recurrence-free survival (RFS) and overall survival (OS) rates were calculated from the date of surgery to the detection of recurrent tumors or to the date of the last follow-up visit, respectively.

### Statistical analysis

Statistical analysis was performed using SPSS for Windows (version 22.0, IBM., Chicago, IL, USA). Intergroup differences in categorical data were assessed for significance using the chi-squared test (2-sided) or Fisher’s exact test. Kaplan–Meier curves were used to estimate cumulative OS and RFS rates, and curves for different patient groups were compared using the log-rank test.

Univariate analysis was used to identify factors associated with the prognosis of HCC patients after liver resection. Statistically significant (*P* < 0.05) variables were then subjected to multivariate Cox analysis to identify independent predictors of OS and RFS. Differences associated with *P* < 0.05 were considered statistically significant.

## Results

### Patient characteristics

Of the 921 patients initially considered, 210 were excluded because (1) they developed potential metastatic disease prior to surgery (*n* = 39), (2) underwent radiofrequency ablation or transcatheter arterial chemoembolization prior to surgery (*n* = 22), or (3) died within one month after surgery or were followed up for less than one month (*n* = 149). Hence, 711 patients met the eligibility criteria and were ultimately enrolled in this retrospective study.

Patients were divided into two groups according to the preoperative serum AFP levels: a low AFP group (*n* = 381; AFP ≤ 400 ng/mL) or a high AFP group (*n* = 300; AFP > 400 ng/mL)] [6]. They were also divided into two groups according to preoperative serum CA19-9 levels: a low CA19-9 group (CA19-9 ≤ 37 U/mL) or a high CA19-9 group (CA19-9 > 37 U/mL)] [11].

The high AFP group had significantly larger tumors, a higher rate of poorly differentiated tumors, and higher aspartate aminotransferase (AST) values than the low AFP group. High AFP levels were also associated with an incomplete tumor capsule, microvascular invasion (MVI), and Barcelona Clinic Liver Cancer (BCLC) C stage.

In contrast to AFP, CA19-9 levels ≤ 37 U/mL were significantly associated with higher values of alanine aminotransferase (ALT), AST, serum albumin, Child–Pugh score, and total bilirubin (TBil), as well as with positive hepatitis B surface antigen (HBsAg) and liver cirrhosis (Table 1).

**Table 1:** Clinicopathological characteristics of patients with hepatocellular carcinoma treated with hepatectomy and enrolled in the study

Variable	Patients (*n* = 711)		*P*	Patients (*n* = 711)		*P*
	AFP ≤ 400 ng/mL (*n* = 381)	AFP > 400 ng/mL (*n* = 330)		CA19-9 ≤ 37 U/mL (*n* = 552)	CA19-9 > 37 U/mL (*n* = 159)	
Sex			0.003			0.485
Male	339	268		474	133	
Female	42	62		78	26	
Age			0.008			0.781
≤60 years	300	285		453	132	
>60 years	81	45		99	27	
Tumor size						
<5 cm	141	82	<0.001	179	44	0.255
≥5 cm	240	248		373	115	
Number of tumors						
<2	271	225	0.394	389	107	0.442
≥2	110	105		163	52	
Tumor capsule			0.006			0.114
Complete	314	244		426	132	
Incomplete	67	86		126	27	
MVI			<0.001			0.469
Negative	189	115		240	64	
Positive	192	215		312	95	
BCLC stage			<0.001			0.058
A–B	289	189		381	97	
C	92	141		171	62	
Edmonson grade			<0.001			0.502
I–II	340	258		467	131	
III–IV	41	72		85	28	
HBsAg			0.141			<0.001
Negative	57	37		89	5	
Positive	324	293		463	154	
Liver cirrhosis			0.789			0.017
No	226	199		343	82	
Yes	155	131		209	77	
Child–Pugh score			0.082			0.142
A	369	326		542	153	
B	12	4		10	6	
Serum albumin			0.752			<0.001
<35 g/L	48	39		54	33	
≥35 g/L	333	291		498	126	
ALT			0.423			<0.001
≤40 U/L	236	214		383	67	
>40 U/L	145	116		169	92	
AST			0.024			<0.001
≤40 U/L	225	167		336	56	
>40 U/L	156	163		216	103	
TBil			0.385			0.008
≤17.1 μmol/mL	297	266		449	114	
>17.1 μmol/mL	84	64		103	45	

*MVI* microvascular invasion, *BCLC* Barcelona Clinic Liver Cancer Staging System, *HBsAg* hepatitis B surface antigen, *ALT* alanine aminotransferase, *AST* aspartate aminotransferase, *TBil* total bilirubin

### Survival based on AFP level, CA19-9 level, or their combination

**Fig. 1:**
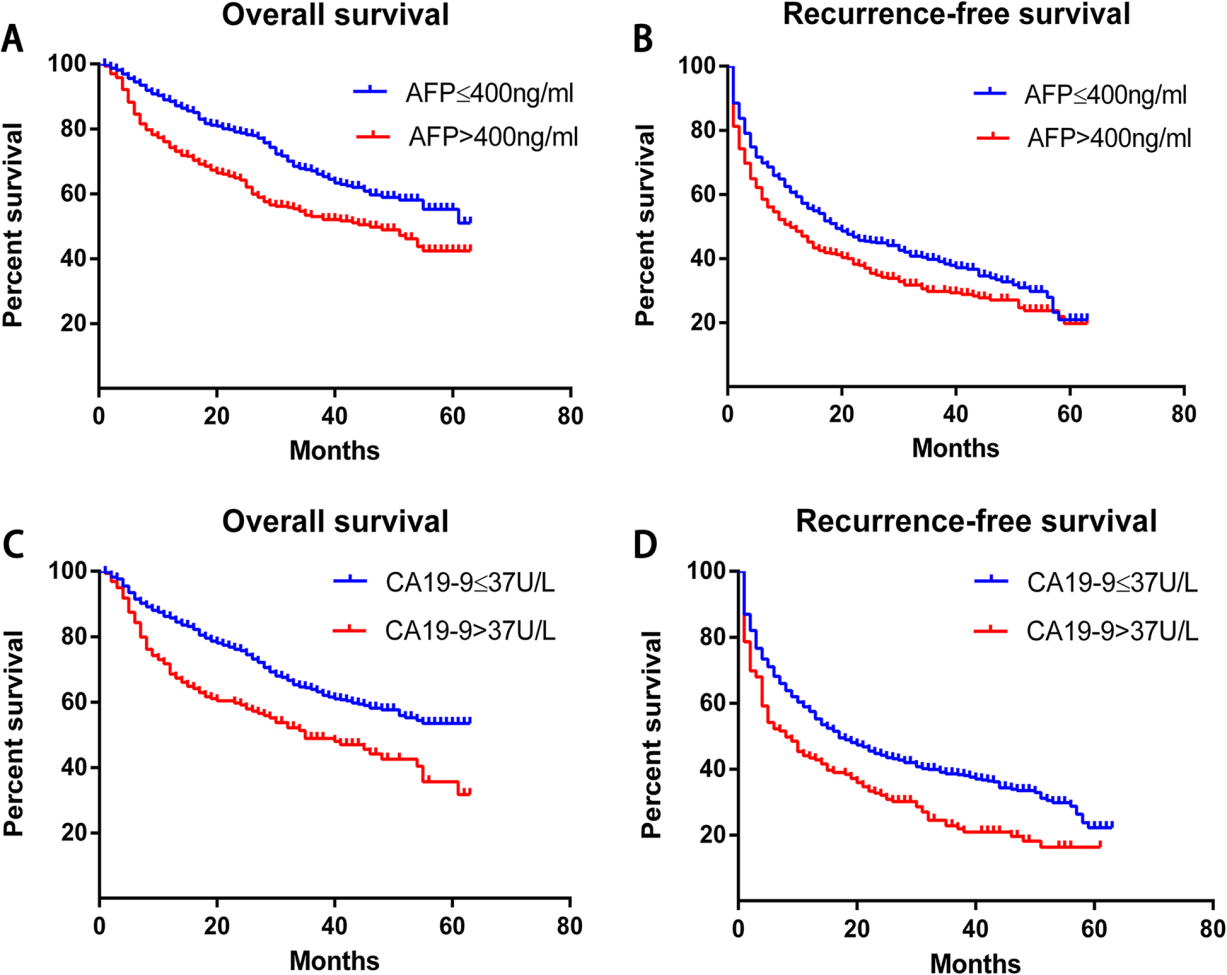
Dependence of **A**, **C** recurrence-free survival and **B**, **D** overall survival of patients with hepatocellular carcinoma after hepatectomy on preoperative serum levels of **A**, **B** α-fetoprotein (AFP) or **C**, **D** carbohydrate antigen 19-9 (CA19-9)

We assessed the OS and RFS rates of patients with different preoperative serum AFP or CA19-9 levels. The 5-year OS and RFS rates of the low AFP group (≤ 400 ng/mL) were significantly higher than those of the high AFP group (> 400 ng/mL) [5-year OS: 50.9 vs. 42.4%, Fig. 1A; 5-year RFS: 20.9 vs. 19.4%, Fig. 1B), suggesting that low preoperative serum AFP levels favor patient survival. In addition, patients with preoperative CA19-9 > 37 U/mL had a significantly worse prognosis than those with CA19-9 ≤ 37 U/mL (5-year OS: 31.7 vs. 53.4%; 5-year RFS: 16.3 vs. 22.2%) (Fig. 1C and D).

**Fig. 2:**
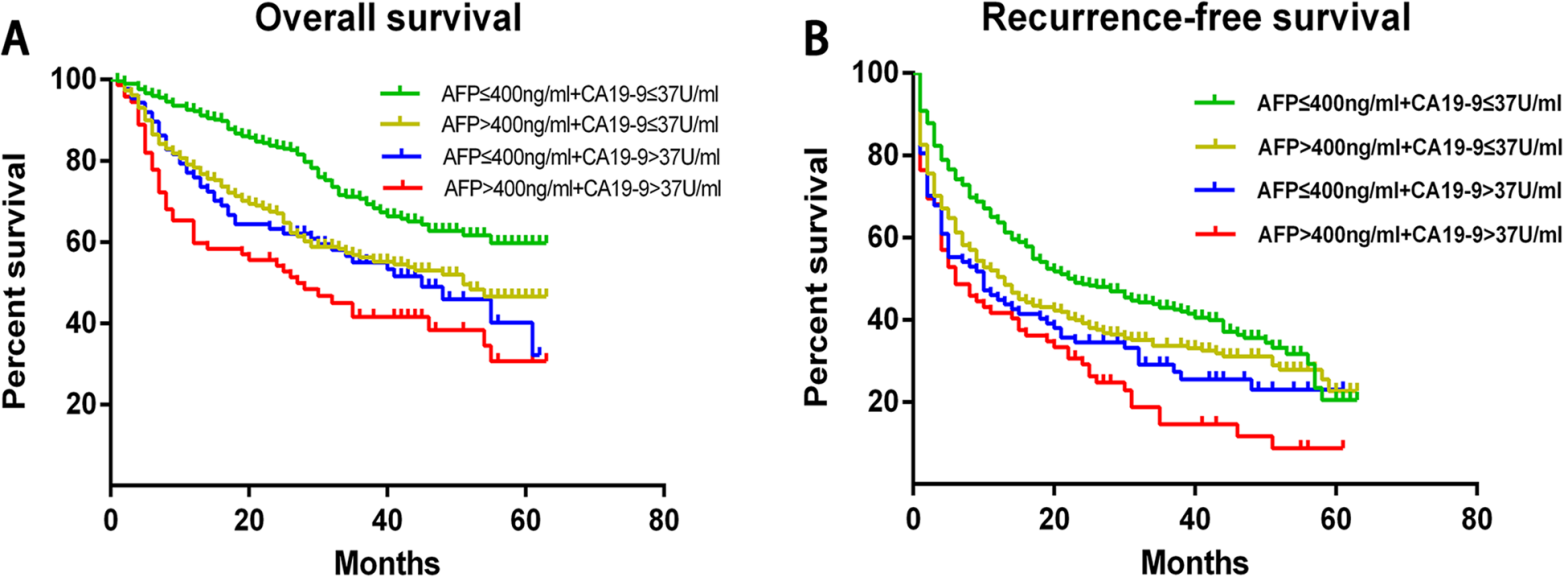
**A** Overall survival and **B** recurrence-free survival of patients stratified by preoperative serum levels of α-fetoprotein (AFP) and carbohydrate antigen 19-9 (CA19-9). Group 1: *n* = 294, green curve; group 2: *n* = 258, yellow curve; group 3: *n* = 87, blue curve; group 4: *n* = 72, red curve. **A** P1–2 < 0.001, P2–3 = 0.601, P1–3 = 0.001, P3–4 < 0.001, P1–4 < 0.001, P2–4 = 0.018); **B** P1–2 = 0.02, P2–3 = 0.334 P1–3 = 0.005, P3–4 = 0.138, P1–4 < 0.001, P2–4 = 0.01

The enrolled patients were also divided into four groups based on different combinations of preoperative AFP and CA19-9 levels (Fig. 2). Patients in group 1 (*n* = 294) had low AFP (≤ 400 ng/mL) and CA19-9 (≤ 37 U/mL) levels, patients in group 2 (*n* = 258) had high AFP (> 400 ng/mL) and low CA19-9 (≤ 37 U/mL), patients in group 3 (*n* = 87) had low AFP (≤ 400 ng/mL) and high CA19-9 (> 37 U/mL), and patients in group 4 (*n* = 72) had high levels of both AFP (> 400 ng/mL) and CA19-9 (> 37 U/mL) (Table 2).

**Table 2:** Preoperative clinicopathological data of enrolled patients, stratified based on preoperative serum levels of AFP and CA19-9

Variable	AFP ≤ 400 ng/mL and CA19-9 ≤ 37 U/mL (*n* =294)	AFP > 400 ng/mL and CA19-9 ≤ 37 U/mL (*n* = 258)	AFP ≤ 400 ng/mL and CA19-9 > 37 U/mL (*n* = 87)	AFP > 400ng/mL and CA19-9 > 37 U/mL (*n* = 72)	*P*
Sex					0.005
Male	267	207	72	61	
Female	27	51	15	11	
Age					0.067
≤60 years	231	222	69	63	
>60 years	63	36	18	9	
Tumor size					0.003
<5 cm	111	68	30	14	
≥5 cm	183	190	57	58	
Number of tumors					0.617
<2	213	176	58	49	
≥2	81	82	29	23	
Tumor capsule					0.019
Complete	239	187	75	57	
Incomplete	55	71	12	15	
MVI					0.001
Negative	148	92	41	23	
Positive	146	166	46	49	
BCLC stage					<0.001
A–B	227	154	62	35	
C	67	104	25	37	
Edmonson grade					0.001
I–II	265	202	75	56	
III–IV	29	56	12	16	
HBsAg					<0.001
Negative	52	37	5	0	
Positive	242	221	82	72	
Liver cirrhosis					0.120
No	182	161	44	38	
Yes	112	97	43	34	
Child–Pugh score					0.090
A	287	255	82	71	
B	7	3	5	1	
Serum albumin					0.002
<35 g/L	28	26	20	13	
≥35 g/L	266	232	67	59	
ALT					<0.001
≤40 U/L	203	180	33	34	
>40 U/L	91	78	54	38	
AST					<0.001
≤40 U/L	192	144	33	23	
>40 U/L	102	114	54	49	
TBil					0.053
≤17.1 μmol/mL	236	213	61	53	
>17.1 μmol/mL	58	45	26	19	

*MVI* microvascular invasion, *BCLC* Barcelona Clinic Liver Cancer Staging System, *HBsAg* hepatitis B surface antigen, *ALT* alanine aminotransferase, *AST* aspartate aminotransferase, *TBil* total bilirub

The 5-year OS rates of groups 1–4 were 59.7%, 46.6%, 32.1%, and 30.7%, respectively, while the respective 5-year RFS rates reached 23.4%, 22.6%, 22.9%, and 18.7%. Thus, the patients in group 4 showed the worst prognosis among the four groups.

**Fig. 3:**
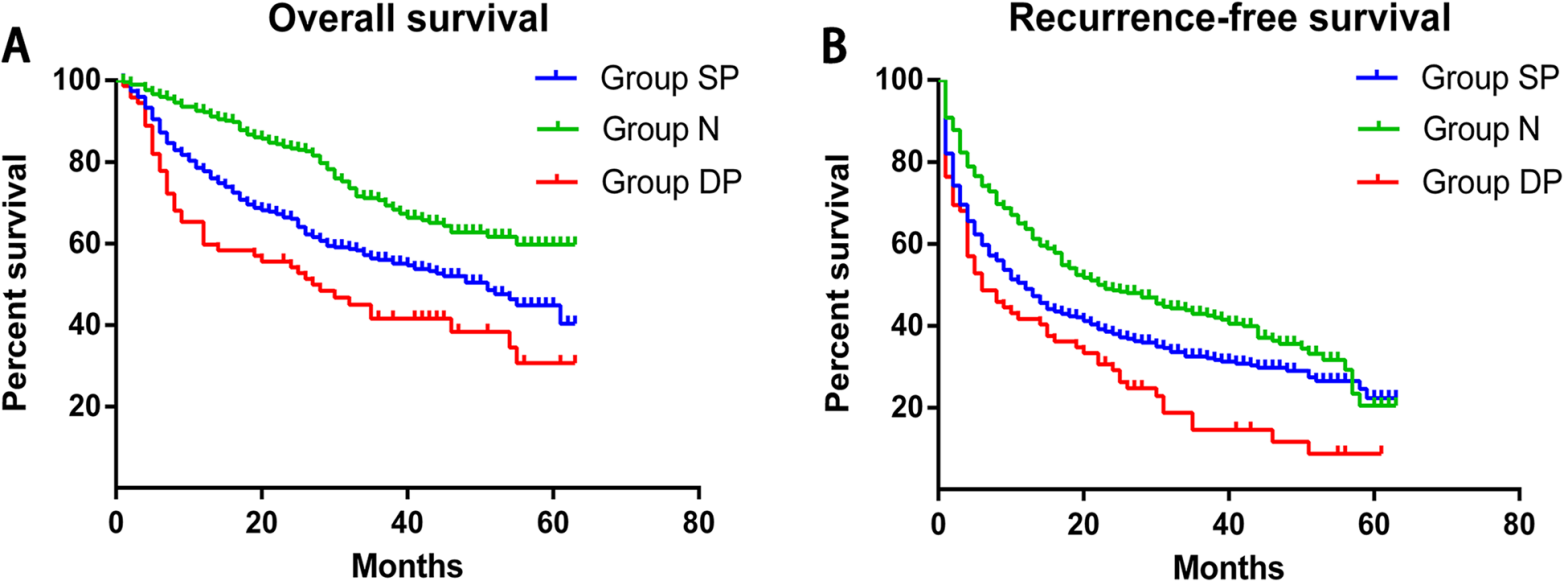
(**A**) Overall survival and (**B**) recurrence-free survival rates of patients stratified by tumor marker type (see the “Results” section): negative (N, green curve), single positive (SP, blue curve), and double positive (DP, red curve). **A** For 5-year OS rates: PN–SP < 0.001, PN–DP < 0.001, PSP–DP = 0.022. **B** For 5-year RFS rates: PN–SP = 0.004, PN–DP < 0.001, PSP–DP = 0.004)

Based on these results, we collapsed the four groups into three tumor markers types (TMTs): the negative (N) group (*n* = 294; group 1), single positive (SP) group (*n* = 345; groups 2 and 3), and double positive (DP) group (*n* = 72; group 4). The 5-year OS rates of the N, SP, and DP groups were 59.7%, 40.3%, and 30.7%, respectively, while the corresponding 5-year RFS rates were 20.5%, 22.3%, and 8.7% (Fig. 3). This confirmed that patients with high levels of both AFP and CA19-9 (DP group) had the worst prognosis of HCC among the three patient groups.

### Survival predictors

The three groups and their clinicopathological data were then analyzed by multiple Cox regression models in which OS and RFS rates were covariates. A total of 18 variables that could affect the OS or RFS of HCC patients were subjected to univariate analysis: age (≤ 60 or > 60 years), sex (male or female), tumor size (≤ 5 or > 5 cm), number of tumors (single or multiple), tumor capsule (incomplete or complete), MVI (positive or negative), BCLC stage (A–B or C), Edmonson grading (I–II or III–IV), HbsAg (positive or negative), liver cirrhosis (yes or no), Child–Pugh class (A or B), serum albumin (≤ 35 or > 35g/L), ALT (≤ 40 or > 40 U/L), AST (≤ 40 or > 40 U/L), TBil (≤ 17.1 or > 17.1 μmol/mL), AFP (≤ 400 or > 400 ng/mL), CA19-9 (≤ 37 or > 37 U/mL), and TMT (N, SP, or DP).

Our analysis revealed that 11 variables were significant predictors for both OS and RFS: large tumor size (> 5 cm), multiple tumor number (≥ 2), incomplete tumor capsule, positive MVI, BCLC-C stage, Edmonson grading III–IV, ALT > 40U/L, AST > 40 U/L, AFP > 400 ng/mL, CA19-9 > 37 U/mL, and TMT (DP). Further multivariate Cox analysis showed that large tumor size, multiple number of tumors, incomplete tumor capsule, positive MVI, BCLC-C stage, and high CA19-9 levels were independent predictors for OS and RFS, whereas high AST level was an independent predictor only for OS (Table 3).

**Table 3:** Uni- and multivariate analyses to identify predictors of recurrence-free and overall survival of patients with hepatocellular carcinoma after hepatectomy

Variable	Recurrence-free survival				Overall survival			
	Univariate analysis	*P*	Multivariate analysis	*P*	Univariate analysis	*P*	Multivariate analysis	*P*
Sex (female)	0.861 (0.666–1.113)	0.253			0.758 (0.540–1.065)	0.110		
Age (>60 years)	0.876 (0.695–1.105)	0.263			0.976 (0.730–1.304)	0.868		
Tumor size (>5 cm)	1.822 (1.483–2.238)	<0.001	1.303 (1.038–1.635)	**0.022**	2.537 (1.902–3.385)	<0.001	1.409 (1.027–1.934)	**0.034**
Number of tumors (multiple)	1.491 (1.236–1.797)	<0.001	1.421 (1.175–1.718)	**<0.001**	1.532 (1.215–1.932)	<0.001	1.509 (1.192–1.923)	**0.001**
Tumor capsule (incomplete)	1.556 (1.267–1.910)	<0.001	1.292 (1.044–1.599)	**0.019**	1.927 (1.510–2.459)	<0.001	1.518 (1.174–1.961)	**0.001**
MVI (positive)	1.622 (1.349–1.950)	<0.001	1.333 (1.095–1.623)	**0.004**	2.173 (1.701–2.774)	<0.001	1.589 (1.222–2.066)	**0.001**
BCLC stage (C)	2.209 (1.840–2.651)	<0.001	1.680 (1.309–2.155)	**<0.001**	3.452 (2.755–4.326)	<0.001	2.239 (1.651–3.036)	**<0.001**
Edmonson grade (III–IV)	1.841 (1.467–2.311)	<0.001	0.954 (0.720–1.264)	0.741	2.727 (2.100–3.540)	<0.001	1.003 (0.727–1.384)	0.985
HbsAg (positive)	1.217 (0.928–1.594)	0.155			0.933 (0.675–1.287)	0.671		
Liver cirrhosis (yes)	1.066 (0.890–1.277)	0.486			1.009 (0.802–1.268)	0.942		
Child–Pugh score (B)	1.256 (0.708–2.227)	0.436			1.893 (1.037–3.456)	0.038	1.174 (0.624–2.208)	0.618
Serum albumin (>35 g/L)	0.850 (0.649–1.112)	0.236			0.598 (0.440–0.812)	0.001	0.841 (0.606–1.168)	0.301
ALT (>40 U/L)	1.266 (1.055–1.519)	0.011	0.977 (0.785–1.216)	0.837	1.360 (1.084–1.707)	0.008	0.963 (0.740–1.259)	0.781
AST (>40 U/L)	1.595 (1.334–1.906)	<0.001	1.233 (0.990–1.537)	0.061	2.019 (1.610–2.532)	<0.001	1.405 (1.075–1.838)	**0.013**
TBil (>17.1 μmol/mL)	0.889 (0.709–1.114)	0.306			0.965 (0.728–1.280)	0.804		
AFP (>400 ng/mL)	1.267 (1.061–1.514)	0.009	1.051 (0.850–1.300)	0.644	1.558 (1.245–1.951)	<0.001	1.308 (0.995–1.720)	0.054
CA19-9 (>37 U/mL)	1.463 (1.193–1.794)	<0.001	1.393 (1.038–1.870)	**0.027**	1.665 (1.301–2.132)	<0.001	1.717 (1.188–2.483)	**0.004**
Tumor marker type (double positive)	1.608 (1.229–2.104)	0.001	1.027 (0.683–1.546)	0.895	1.875 (1.362–2.581)	<0.001	1.259 (0.763–2.791)	0.366

*MVI* microvascular invasion, *BCLC* Barcelona Clinic Liver Cancer Staging System, *HBsAg* hepatitis B surface antigen, *ALT* alanine aminotransferase, *AST* aspartate aminotransferase, *TBil* total bilirubin, *AFP* α-fetoprotein, *CA19-9* carbohydrate antigen 19-9

## Discussion

In this retrospective study, we showed that AFP and CA19-9, as single tumor markers, are associated with the OS and RFS of HCC patients after radical hepatectomy, consistent with the results of previous studies. In order to improve the prognostic efficiency for postoperative HCC recurrence, we assessed the combination of AFP and CA19-9 as a single prognostic indicator, as sensitivity and specificity in tumor diagnosis can be significantly increased by combining two or more serum tumor markers [15, 16]. In particular, we found that patients with high preoperative serum AFP (> 400 ng/mL) and CA19-9 (> 37 U/mL) levels had a worse prognosis than those with other TMTs. In addition to AFP and CA19-9, univariate and multivariate regression analysis identified various preoperative clinicopathological features significantly associated with the OS and RFS of HCC patients.

Continuous advances in the diagnosis and treatment of HCC have significantly improved the short-term treatment of HCC. However, the long-term survival of HCC is still unsatisfactory, and the postoperative recurrence rate remains considerably high [3, 17]. Therefore, further research is needed to clarify the factors affecting the prognosis of HCC, improve the survival time, and reduce the overall postoperative recurrence rate of HCC by establishing a more accurate prognosis assessment system [18].

In addition to conventional clinicopathological indicators, AFP has also been identified as a prognostic factor for postoperative HCC [5, 13, 19]. Recent European guidelines suggest that AFP levels greater than 400 ng/mL are associated with poor prognosis [6]. Although our data confirmed the prognostic value of this AFP threshold, serum AFP was not high in all patients and those with high AFP levels differed substantially in prognosis, reflecting the heterogeneity of HCC.

CA19-9 is commonly used in clinical practice, especially for the diagnosis of adenocarcinoma, and has been recently reported as a valuable prognostic marker for HCC [12, 20, 21]. The results of a prospective study suggested that serum CA19-9 levels ≥ 100 U/mL are an independent predictor of poor OS in HCC patients, which probably reflects the activity of progenitor-like cells in the non-tumor liver [22]. In contrast, a retrospective study reported that preoperative CA19-9 levels > 27 U/mL are associated with poor prognosis after resection [12], while a study of 11,096 patients demonstrated that CA19-9 levels > 37 U/mL accurately discriminate pancreatic cancer from benign pancreatic diseases [23]. Our own work further supports a correlation between CA19-9 levels > 37 ng/mL and poor prognosis of HCC [11]. In addition, we tried to find the optimal CA19-9 threshold for DFS and OS in HCC patients by ROC curve and found that the test power of the optimal threshold CA19-9 > 30.21 U/mL was not significantly better than CA19-9 > 37 U/mL, considering that CA19-9 < 37 U/mL is also the normal medical reference value range, which may be more convenient for clinical application, we finally used this concentration as the cut-off in the present study.

How elevated CA19-9 leads to poor prognosis of HCC patients is still unclear. CA19-9 may change the molecular microenvironment and promote the proliferation, invasion, and metastasis of cancer cells by participating in cell growth and differentiation, signal transmission, apoptosis, white blood cell aggregation, immune regulation, and other physiological processes [24]. AFP and CA19-9 may influence the tumor biological characteristics of HCC through different mechanisms, with similar consequences for prognosis. AFP can block the RA-RAR signaling pathway, thereby promoting tumor cell growth and disrupting the onward transduction of apoptotic signaling by binding with caspase-3, while it can activate the PI3K/AKT pathway by causing dysfunction in the phosphatase and tensin homolog, leading to irregular HCC cell proliferation [25–27]. Moreover, HCC patients with high AFP serum levels (> 300 ng/mL) and positive staining for the epithelial cell adhesion molecule EpCAM show significantly higher microvessel density and tissue expression of vascular endothelial growth factor, which may be related to tumor angiogenesis [28]. CA19-9 is associated with the severity of liver failure [29], which is also supported by our results as well because high CA19-9 is associated with higher bilirubin and transaminase levels in our study. Some patients with HCC, especially those with large tumors, who have cirrhosis, often die of liver failure rather than tumor recurrence. Therefore, this is also one of the reasons for the poor postoperative prognosis of HCC patients caused by elevated CA19-9.

CA19-9 is often associated with tumors of epithelial origin, such as cholangiocarcinoma and pancreatic cancer [30, 31]. The CA19-9 level of the patients of pancreatic cancer can even reach 1000U/ml [32], which is much higher than that of HCC. Therefore, some studies believe that HCC with elevated CA19-9 has certain biological characteristics of intrahepatic cholangiocarcinoma [33, 34]. Although some HCC tumor cells which were named dual-phenotype hepatocellular carcinoma (DPHCC) showed the phenotypic characteristics of intrahepatic cholangiocarcinoma, they still belonged to HCC morphologically. DPHCC is a novel surgicopathologic entity that has a highly aggressive behavior and worse postoperative prognosis than pure HCC [11, 33]. Therefore, CA19-9 may be potentially related to DPHCC.

Although our current study supports that the combination of AFP and CA19-9 can greatly improve the prediction of post-hepatectomy prognosis for HCC patients, our results should be interpreted carefully due to several limitations such as the lack of additional serum markers and short follow-up of the enrolled patients. Multivariate Cox analysis showed that TMT was not an independent factor for the survival of patients. This implies that TMT by itself in sufficient for predicting prognosis. Instead, TMT should be considered together with several other clinicopathological factors, such as BCLC stage, tumor size, and Edmonson grade. Nevertheless, patients with high AFP and CA19-9 levels may need to be monitored more carefully and analyzed in greater depth, such as through postoperative pathological examination, in order to fully understand their tumor characteristics and ensure the detection of other recurrence risk factors such as MVI. This may help improve the treatment and management of patients at higher risk of HCC recurrence after surgery.

## Conclusion

Our study indicates that AFP and CA19-19 could efectively predict the prognosis of patients with HCC after surgery. The predictive ability of combined the two biomarkers is relatively promising, and after more extensive evaluation and broadened analysis from different populations, it may assist clinicians in a more precise prediction of prognosis.

## Data Availability

The raw data of this manuscript are available upon reasonable request from the corresponding author.

## Abbreviations

HCC: Hepatocellular carcinoma
AFP: α-fetoprotein
CA19-9: Carbohydrate antigen 19-9
DP: AFP and CA19-9 double positive
SP: AFP or CA19-9 single positive
N: AFP and CA19-9 negative
OS: Overall survival
RFS: Recurrence-free survival
AST: Aspartate aminotransferase
ALT: Alanine aminotransferase
TBil: Total bilirubin
HBsAg: Hepatitis B surface antigen
MVI: Microvascular invasion
BCLC: Barcelona Clinic Liver Cancer
TMTs: Tumor markers types

## References

[1] Siegel RL, Miller KD, Fuchs HE, Jemal A (2021). Cancer statistics, 2021. CA Cancer J Clin.

[2] Zhong JH, Rodríguez A, Ke Y, Wang YY, Wang L, Li LQ (2015). Hepatic resection as a safe and effective treatment for hepatocellular carcinoma involving a single large tumor, multiple tumors, or macrovascular invasion. Medicine (Baltimore).

[3] Bureau of Medical Administration, National Health Commission of the People's Republic of China (2020). Guidelines for diagnosis and treatment of primary liver cancer in china (2019 edition). J Clin Hepatol.

[4] Luo P, Wu S, Yu Y, Ming XL, Li S, Zuo XL (2019). Current Status and Perspective Biomarkers in AFP Negative HCC: Towards Screening for and Diagnosing Hepatocellular Carcinoma at an Earlier Stage. Pathol Oncol Res.

[5] Jiang JH, Wang KX, Zhu JY, Yang PP, Guo Z, Ma SL (2017). Comparison of hepatectomy with or without hepatic inflow occlusion in patients with hepatocellular carcinoma: a single-center experience. Minerva Med.

[6] European Association for the Study of the Liver Office. EASL-EORTC clinical practice guidelines: management of hepatocellular carcinoma. J Hepatol. 2012;56(4):908–43. 10.1016/j.jhep.2011.12.001 22424438

[7] Scarà S, Bottoni P, Scatena R (2015). CA19-9: Biochemical and Clinical Aspects. Adv Exp Med Biol.

[8] Xu HX, Liu L, Xiang JF, Wang WQ, Qi ZH, Wu CT (2017). Postoperative serum CEA and CA125 levels are supplementary to perioperative CA19-9 levels in predicting operative outcomes of pancreatic ductal adenocarcinoma. Surgery..

[9] Ushigome M, Shimada H, Miura Y, Yoshida K, Kaneko T, Koda T (2019). Changing pattern of tumor markers in recurrent colorectal cancer patients before surgery to recurrence: serum p53 antibodies, CA19-9 and CEA. Int J Clin Oncol.

[10] Jo JC, Ryu MH, Koo DH, Ryoo BY, Kim HJ, Kim TW (2013). Serum CA19-9 as a prognostic factor in patients with metastatic gastric cancer. Asia Pac J Clin Oncol.

[11] Zhang J, Qi YP, Ma N, Lu F, Gong WF, Chen B (2020). Overexpression of Epcam and CD133 Correlates with Poor Prognosis in Dual-phenotype Hepatocellular Carcinoma. J Cancer.

[12] Chen YL, Chen CH, Hu RH, Ho MC, Jeng YM (2013). Elevated preoperative serum CA19-9 levels in patients with hepatocellular carcinoma is associated with poor prognosis after resection. ScientificWorldJournal..

[13] Ma WJ, Wang HY, Teng LS (2013). Correlation analysis of preoperative serum alpha-fetoprotein (AFP) level and prognosis of hepatocellular carcinoma (HCC) after hepatectomy. World J Surg Oncol.

[14] Zhou L, Rui JA, Wang SB, Chen SG, Qu Q (2018). Carbohydrate Antigen 19-9 Increases the Predictive Efficiency of α-Fetoprotein for Prognosis of Resected Hepatocellular Carcinoma. Am Surg.

[15] He CZ, Zhang KH, Li Q, Liu XY, Hong Y, Lv NH (2013). Combined use of AFP, CEA, CA125 and CAl9-9 improves the sensitivity for the diagnosis of gastric cancer. BMC Gastroenterol.

[16] Zhou J, Zhu Y, Li Y, Liu K, He F, Xu S (2021). Combined detection of circulating tumor cells, α-fetoprotein heterogene-3 and α-fetoprotein in the early diagnosis of HCC for the prediction of efficacy, prognosis, recurrence after microwave ablation. Infect Agent Cancer.

[17] Vogel A, Saborowski A (2020). Current strategies for the treatment of intermediate and advanced hepatocellular carcinoma. Cancer Treat Rev.

[18] Nishida N (2020). Long-term prognosis and management of hepatocellular carcinoma after curative treatment. Clin Mol Hepatol.

[19] Kang SH, Kim DY, Jeon SM, Ahn SH, Park JY, Kim SU (2012). Clinical characteristics and prognosis of hepatocellular carcinoma with different sets of serum AFP and PIVKA-II levels. Eur J Gastroenterol Hepatol.

[20] Lu LH, Zhang YF, Wei W, Shi M, Guo RP (2017). Preoperative Carbohydrate Antigen 19-9: Its Neglected Role in Alpha-Fetoprotein-Negative Hepatocellular Carcinoma Patients. J Gastrointest Surg.

[21] Ding M, Zhao X, Zhao M, Shi YP, Wang T, Cui D (2020). Prognostic Nomogram for Patients with Hepatocellular Carcinoma After Thermal Ablation. Cardiovasc Intervent Radiol.

[22] Hsu CC, Goyal A, Iuga A, Krishnamoorthy S, Lee V, Verna E (2015). Elevated CA19-9 Is Associated With Increased Mortality In A Prospective Cohort Of Hepatocellular Carcinoma Patients. Clin Transl Gastroenterol.

[23] Kim HR, Lee CH, Kim YW, Han SK, Shim YS, Yim JJ (2009). Increased CA19-9 level in patients without malignant disease. Clin Chem Lab Med.

[24] Luo G, Jin K, Deng S, Cheng H, Fan ZY, Gong YT (2021). Roles of CA19-9 in pancreatic cancer: Biomarker, predictor and promoter. Biochim Biophys Acta Rev.

[25] Li MS, Li H, Li CY, Wang SS, Jiang W, Liu ZG (2011). Alpha-fetoprotein: A new member of intracellular signal molecules in regulation of the PI3K/AKT signaling in human hepatoma cell lines. Int J Cancer.

[26] Zhu M, Guo J, Xia H, Li W, Lu Y, Dong X (2015). Alpha-fetoprotein activates AKT/mTOR signaling to promote CXCR4 expression and migration of hepatoma cells. Oncoscience..

[27] Li MS, Li H, Li CY, Guo LY, Liu H, Zhou S (2009). Cytoplasmic alpha-fetoprotein functions as a co-repressor in RA-RAR signaling to promote the growth of human hepatoma Bel 7402 cells. Cancer Lett.

[28] Galle PR, Foerster F, Kudo M, Chan SL, Llovet JM, Qin SK (2019). Biology and significance of alpha-fetoprotein in hepatocellular carcinoma. Liver Int.

[29] Halme L, Kärkkäinen P, Isoniemi H, Mäkisalo H, von Bogulawski K, Höckerstedt K (1999). Carbohydrate 19-9 antigen as a marker of non-malignant hepatocytic ductular transformation in patients with acute liver failure. Scand J Gastroenterol.

[30] Li H, Feng Y, Liu C, Li J, Li J, Wu H (2021). Importance of Normalization of Carbohydrate Antigen 19-9 in Patients With Intrahepatic Cholangiocarcinoma. Front Oncol.

[31] Li M, Dong ZY, Zhang XF, Xue SH, Wang B (2022). Analysis of levels and Clinical value of CA19-9, NLR and SIRI in patients with Pancreatic Cancer with different Clinical Features. Cell Mol Biol (Noisy-le-grand).

[32] Liu L, Xu H, Wang W, Wu C, Chen Y, Yang J (2015). A preoperative serum signature of CEA+/CA125+/CA19-9 ≥ 1000 U/mL indicates poor outcome to pancreatectomy for pancreatic cancer. Int J Cancer.

[33] Lu XY, Xi T, Lau WY, Dong H, Zhu Z, Shen F (2011). Hepatocellular carcinoma expressing cholangiocyte phenotype is a novel subtype with highly aggressive behavior. Ann Surg Oncol.

[34] Wang H, Cong WM (2017). Research progress on clinicopathology in dual-phenotype hepatocellular carcinoma. Chin J Clin Oncol.

